# Data from the numerical analysis of radial and tangential leakage of gas in scroll compressors

**DOI:** 10.1016/j.dib.2020.105197

**Published:** 2020-01-28

**Authors:** Evandro L.L. Pereira, Vitor M. Braga, Cesar J. Deschamps

**Affiliations:** POLO Research Laboratories for Emerging Technologies in Cooling and Thermophysics, Federal University of Santa Catarina, CEP 88040-900, Florianopolis, SC, Brazil

**Keywords:** Scroll compressor, Radial leakage, Tangential leakage, Numerical analysis, CFD

## Abstract

The data presented herein are related to the simulations and correlations reported in the article entitled “Numerical analysis and correlations for radial and tangential leakage of gas in scroll compressors” [1]. The numerical simulations were carried out using the ANSYS FLUENT 12.1.4 CFD code, which is based on the finite volume method. Details of the numerical modeling setup and data collection are provided to allow reproducibility. The datasets include predicted values of radial and tangential leakage carried out using ANSYS FLUENT 12.1.4 CFD code for different gases, operating conditions and geometries expressed as dimensionless parameters. The scripts in C++ for the correlations of radial and tangential leakages developed based on the numerical predictions are also provided together with experimental and numerical data used to validate the correlations.

**Table:** Specifications Table

Subject	Mechanical Engineering
Specific subject area	Fluid dynamics, Leakage, Refrigeration
Type of data	Figures, tables, datasets, graphs, computational algorithm.
How data were acquired	The ANSYS FLUENT v. 12.1.4 CFD code to predict radial and tangential leakage of gas. The software Eureqa to develop correlations for radial and tangential leakages.
Data format	Raw and analyzed
Parameters for data collection	The geometric models and boundary conditions adopted for the numerical simulation are those typically found in scroll compressors. Dimensional analysis was carried out to reduce the number of variables in the problem.
Description of data collection	The data for the numerical simulations was collected using ANSYS FLUENT v. 12.1.4 CFD code, which adopts the finite volume method. Several simulations were conducted with different boundary conditions to explore the influence of the dimensionless parameters over the tip and radial leakage in scroll compressors. The data for the developed correlation were collected using a script written in C++.
Data source location	Federal University of Santa Catarina, Brazil
Data accessibility	Data is within this article.
Related research article	E.L.L. Pereira, C.J. Deschamps, Numerical Analysis and Correlations for Radial and Tangential Leakage of Gas in Scroll Compressors. Int. J. Refrigeration. 110 (2020) 239–247. https://doi.org/10.1016/j.ijrefrig.2019.11.002

**Table:** 

**Value of Data** • The data are useful for the reproduction of the numerical simulations in other research laboratories. • The data can also be used to test different parameters, such as turbulence models, grid refinement and discretization schemes. • Correlations for radial and tangential leakage are given in form of C++ scripts suitable for implementation in lumped simulation models of scroll compressors. • The data and correlations can benefit engineers in the development of scroll compressors. • The data can be further used to estimate measurement uncertainty of leakage.

## 1 Data

The data presented in this article is associated with predictions of radial and tangential leakage of gas in the tip (radial leakage) and flank (tangential leakage) clearances of scroll compressors. The numerical solution procedure is described, and the criteria and equations adopted to generate the computational mesh are also explained.

File 1 contains the dataset of numerical results from the simulations, regarding the influence of the dimensionless parameters P, Π, $\delta_{t}^{*}$, $\delta_{f}^{*}$ and $C^{*}$ on the dimensionless mass flow rate M (see Ref. [1] for details on these dimensionless parameters). Additionally, the dimensionless pressure variation ($p/p_{l})$ along the tip and flank clearances, associated with the radial and tangential leakages, is also given for different values of the aforementioned dimensionless parameters.

File 2 presents the scripts in C++ for the correlations of radial and tangential leakages, which were developed with the software Eureqa [2] based on the numerical dataset of the simulations, along with an example of usage.

Finally, File 3 provides the dataset of experimental data [[3], [4], [5], [6]] and numerical results [7,8] used to validate the correlations for radial and tangential leakage.

## 2 Experimental design, materials, and methods

The numerical simulations were carried out using ANSYS FLUENT v. 12.1.4 CFD code. The simulations and development of correlations for radial and tangential leakage were conducted through the following steps: (i) Setup of the flow geometry; (ii) Setup of the discretization mesh; (iii) Definition of the boundary conditions; (iv) Numerical calculation and sensitivity analysis of predictions regarding discretization mesh and turbulence modeling; (v) Development of correlations with Eureqa [2]; (vi) Writing of program scripts for the correlations; (vii) Validation of the correlations.

**Fig. 1:**
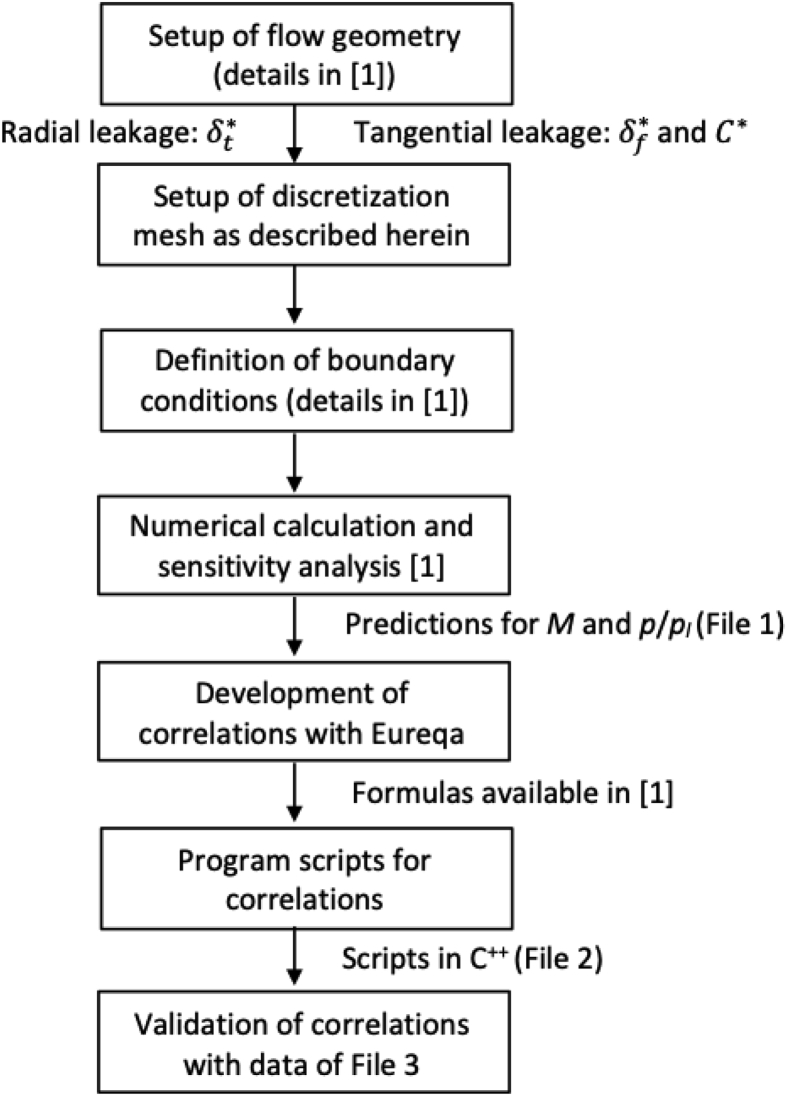
Model schematic of the simulations and development of correlations.

As indicated in Fig. 1, the first step is the input of the geometric data to the CFD model. The dimensionless clearance $\delta_{t}^{*}$ defines the flow geometry of the radial leakage, whereas the dimensionless clearance $\delta_{f}^{*}$ and curvature $C^{*}$ characterize the flow geometry of the tangential leakage. Then, the computational mesh is generated for the simulations following two basic criteria: (i) the values of $y^{+}$ for grid cells adjacent to the solid walls should be close to 1; (ii) the viscous wall region ($y^{+}<30$) should be discretized with at least 10 grid cells. The minimum size of the cell adjacent to the walls ($y_{p}$) is determined by:

(1) $$y_{p}=\frac{y^{+}\mu }{\rho u_{\tau }\;}$$

where the friction velocity $u_{\tau }=U\sqrt{{\bar{C}}_{f}/2}$ is calculated by assuming the following empirical correlation for turbulent flow over a flat plate,

(2) $${\bar{C}}_{f}=\frac{0.037}{Re_{L}^{\frac{1}{5}}}$$

in which the Reynolds number $Re_{L}$ is based on the average length of the chamber at the end of the suction process L=2πa(2πN−π). The characteristic velocity of the flow is given by:

(3) $$U=\frac{{\dot{m}}_{th}}{\rho_{h}\delta w}$$

where the theoretical mass flow rate (${\dot{m}}_{th}$) is calculated for isentropic flow in a nozzle, $\rho_{h}$ is the gas density in the high-pressure side and δ and w represent the gap length and the gap width, respectively.

Next, the number of cells in the direction normal to the wall are calculated:

(4) $$N_{\hat{n}}=2\;\text{int}\left[\frac{\log\left(\frac{r_{0}\left(q_{\hat{n}}-1\right)}{\Delta n_{w}}\right)}{\log q_{\hat{n}}}\right]$$

with the cell growth ratio $q_{\hat{n}}=1.1$ and $\Delta n_{w}=2y_{p}$ the maximum height of the cells adjacent to the wall. The function ‘int’ returns the value rounded to the nearest integer. Additionally, we established a minimum value of $N_{\hat{n}}=20$, assuring proper mesh refinement of the boundary layer for flows of low Reynolds number.

In the tangential direction, the number of cells ($N_{\hat{s}})$ was kept the same for all simulations, with a minimum size calculated by:

(5) $$\Delta s_{w}=\frac{L_{w}\left(q_{\hat{s}}-1\right)}{2q_{\hat{s}}^{N_{\hat{s}}}}$$

where $L_{w}$ is the length of the wall and $q_{\hat{s}}=1.1$ is the cell growth ratio in the tangential direction. In addition to controlling the cell growth ratio, we also ensured that the cell aspect ratio in the region far from the wall is kept below 10:1.

It should be mentioned that the actual values of $y^{+}$ for the flow will not necessarily be equal to the values assumed in Equation (1), due to the simplifications adopted for the friction coefficient ${\bar{C}}_{f}$ and the Reynolds number. Thus, the values of $y^{+}$ must be checked after each simulation, in order to assure that criterion (i) is met.

With the computational mesh defined, the boundary conditions are prescribed as detailed in Ref. [1] and the transport equations are solved via an iterative procedure that is repeated until reaching the specified convergence criterion. After carrying out a sensitivity analysis of the predictions regarding the computational mesh and turbulence modeling, the dimensionless mass flow rate M is evaluated for different values of the dimensionless parameters P, Π, $\delta_{t}^{*}$, $\delta_{f}^{*}$ and $C^{*}$ according to the geometry of interest, i.e., axial clearance in the case of radial leakage and radial clearance in the case of tangential leakage. Such datasets are provided in File 1.

Based on the numerical datasets of File 1, the software Eureqa [2] was then used to develop correlations for the radial and tangential leakage of gas in scroll compressors. The software Eureqa [2] finds several formulas and the most convenient is selected according to criteria of simplicity and accuracy to represent the numerical datasets. In fact, the data in File 1 can be used to develop other alternatives of correlations for leakage by further analysis with Eureqa [2] or different approach.

After selecting the most convenient formulas, scripts in C++ were written for the correlations of radial and tangential leakage. Such scripts, along with an example of usage, are made available in File 2. Finally, the correlations were validated through comparisons with experimental data [[3], [4], [5], [6]] and numerical results [7,8] provided in File 3. It should be mentioned that the scripts in C++ are particularly suitable for implementation in lumped models frequently adopted to simulate and design scroll compressors Moreover, these correlations can also be used to estimate measurement uncertainty of leakage.
